# Breaking self-incompatibility for diploid hybrid potato breeding: advances, mechanisms, and emerging technologies

**DOI:** 10.3389/fpls.2026.1848562

**Published:** 2026-05-21

**Authors:** Yiqian Wang, Tuanrong Lin, Zhen Wang, Longqiu Fan, Yufeng Wang, Xinlei Jiao, Wei Wang, Xiaojie Zong, Junzhi Chen, Yuhe Yin

**Affiliations:** 1 Ulanqab Academy of Agriculture and Forestry Sciences, Inner Mongolia Potato Technology Innovation Center, Ulanqab, Inner Mongolia, China; 2 College of Horticulture and Plant Protection, Inner Mongolia Agricultural University, Hohhot, Inner Mongolia, China; 3 Forestry Workstation, Forestry and Grassland Administration of Ulanqab City, Ulanqab, Inner Mongolia, China

**Keywords:** apomixis, diploid hybrid breeding, genome editing, inbred lines, potato, self-incompatibility, *Sli* gene

## Abstract

Potato (*Solanum tuberosum* L.) is a staple food crop vital to global food security. Conventional tetraploid potato breeding is severely constrained by tetrasomic inheritance, long breeding cycles, low propagation efficiency, and accumulated genetic load. Diploid hybrid breeding based on homozygous inbred lines represents a promising strategy to restructure potato improvement. However, most diploid potatoes exhibit strong gametophytic self-incompatibility, which blocks the development of inbred lines and limits hybrid breeding. In recent years, remarkable progress has been made in elucidating the molecular basis of potato self-incompatibility, including the S-locus, *S-RNase*, *SLF/SFB* genes, and the pivotal S-locus inhibitor gene. Meanwhile, innovative strategies including S-locus inhibitor gene introgression, genome editing, haploid induction and apomixis have facilitated the development of homozygous inbred lines and hybrid breeding and the development of homozygous inbred lines. This mini review systematically summarizes recent advances in the molecular mechanisms of potato self-incompatibility, key technologies to overcome self-incompatibility, and innovative diploid hybrid breeding paradigms. We further propose integrated strategies combining precision genome editing, speed breeding, and apomixis to accelerate hybrid variety development, offering a practical guide for diploid hybrid potato improvement.

## 1 Introduction

Potato (*Solanum tuberosum* L.) is the third most important food crop in the world, providing essential calories and nutrients for global food security (Devaux et al., 2021; FAO, 2024; Song et al., 2024; Gietler et al., 2025). Conventional potato breeding relies on tetraploid cultivars with tetrasomic inheritance, which severely limits genetic analysis and selection efficiency (Jansky and Spooner, 2018; Bethke et al., 2019; Louwaars, 2023; Sun et al., 2025). In addition, tuber propagation leads to high costs, easy transmission of viruses, and slow variety replacement (Paul et al., 2022). To overcome these bottlenecks, diploid hybrid potato breeding has been proposed and rapidly developed, aiming to establish a hybrid seed system similar to rice or maize, using true potato seed (TPS) for production (Lindhout et al., 2011; de Vries et al., 2023). This system can significantly shorten the breeding cycle, improve genetic purity, and facilitate rapid deployment of disease resistance and stress-tolerance traits (Kacheyo, 2024). However, most diploid potatoes display strong gametophytic self-incompatibility (GSI), which prevents self-fertilization and thus hinders the production of homozygous inbred lines (Jing et al., 2025). Beyond self-incompatibility (SI), key bottlenecks also include severe inbreeding depression, high deleterious mutation load, and reduced male and female fertility in diploid germplasm (Wu et al., 2023). The SI response in diploid potatoes is controlled by the polymorphic S-locus, which encodes the female determinant *S-RNase* and male determinant S-locus F-box (*SLF/SFB*) proteins (Wang and Kao, 2012). In recent years, significant breakthroughs have been achieved in cloning key genes (e.g., *Sli*), dissecting regulatory mechanisms of SI, and developing technologies to overcome SI in diploid potatoes. This mini review focuses on recent advances in SI mechanisms, strategies to overcome SI, and innovative diploid hybrid breeding paradigms, and provides perspectives for future research.

## 2 Molecular basis of self-incompatibility in diploid potatoes

### 2.1 *S*-locus and GSI mechanism

Diploid potato SI conforms to the *S-RNase*-dependent GSI system, which is evolutionarily highly conserved throughout the Solanaceae family (Jing et al., 2025; Xue, 2025). GSI is genetically controlled by a single, highly polymorphic genomic region termed the S-locus, which harbors two tightly linked and specifically expressed genes responsible for self- and non-self-recognition (Kao and Tsukamoto, 2004). Key genetic properties include codominance of S-haplotypes, extreme allelic diversity, and long-term maintenance of S-alleles through balancing selection, as well as trans-specific polymorphism. These features render SI a persistent genetic barrier in diploid potato breeding. The female determinant is *S-RNase*, a ribonuclease-encoding gene exclusively expressed in stylar transmitting tissue. The male determinants correspond to a cluster of *SLF/SFB* genes, which are specifically expressed in pollen and pollen tubes (Fujii et al., 2016). The collaborative non-self-recognition model explains compatible pollination: individual *SLF* proteins recognize and detoxify non-self *S-RNase*s, while self *S-RNase*s remain cytotoxic. During pollination, both self and non-self *S-RNase*s enter pollen tubes. In incompatible pollination, self *S-RNase* retains cytotoxic activity and triggers a broad range of cytotoxic and signaling responses, rather than merely causing rRNA degradation. These responses further induce pollen tube depolarization, cytoskeleton disorganization and growth arrest, ultimately resulting in fertilization failure. In compatible (non-self) pollination, individual SLF proteins recognize and interact with non-self *S-RNase*s, assembling functional Skp1–Cullin1–F-box (SCF) E3 ubiquitin ligase complexes. These complexes target non-self *S-RNase*s for ubiquitination and subsequent degradation through the 26S proteasome pathway. As a result, cytotoxicity is neutralized, pollen tubes grow normally toward the ovary, and successful fertilization is achieved (Xue, 2025). This strict self/non-self discrimination mechanism effectively prevents inbreeding and preserves genetic diversity in natural populations. In Solanaceae, SI represents a genetically controlled prezygotic reproductive barrier rather than a simple physiological barrier. Nevertheless, several core mechanistic questions remain unresolved; in particular, the exact action mode and molecular specificity of *SLF/SFB* proteins in recognizing and detoxifying *S-RNase* have not been fully clarified.

### 2.2 *Sli* gene: key determinant for overcoming self-incompatibility

A pivotal breakthrough in diploid potato hybrid breeding was the identification and functional characterization of the S-locus inhibitor (*Sli*) gene, originally derived from self-compatible (SC) accessions of *Solanum chacoense* and subsequently identified in several self-fertile cultivated diploid potato clones (Hosaka and Hanneman, 1998a). *Sli* was mapped to chromosome 12, followed by fine mapping and gene cloning (Hosaka and Hanneman, 1998b). Transgenic complementation confirmed its function in conferring SC, while gene knockout led to the restoration of SI. Molecular studies confirmed that *Sli* encodes a non-S-locus F-box protein that acts as a major broad-spectrum compatibility factor for diverse *S-RNase* alleles (Ma et al., 2021).

By inhibiting the cytotoxic activity of self *S-RNase*s in a pollen-specific manner, *Sli* effectively breaks GSI and enables stable self-fertilization in typically self-incompatible diploid potatoes. Recent mechanistic investigations further revealed that a MITE transposon insertion in the *Sli* promoter region is responsible for conferring pollen-specific expression, but this insertion is not strictly required for self-compatibility in all genetic backgrounds (Eggers et al., 2021; Ma et al., 2021; Zhang et al., 2025). Notably, *Sli* function shows incomplete penetrance: some genotypes carrying *Sli*-associated alleles still exhibit residual SI, indicating dependence on genetic background and additional modifiers. *Sli* homozygosity can be obtained in adapted materials such as M6, but is associated with lethality or segregation distortion in other genetic backgrounds (Hosaka and Hanneman, 1998a; Birhman and Hosaka, 2000; Kaiser et al., 2021). *Sli* serves as a key determinant of self-compatibility and foundational tool for generating homozygous inbred lines.

### 2.3 Other modifiers of self-compatibility

Besides the core *Sli* gene that confers strong SC in potato, multiple additional regulatory genes and pathway components act as modifier factors affecting SI and SC responses. These modifiers act either independently or in coordination with core SI/SC regulators to modulate pollen–pistil communication, pollen tube elongation and downstream self-incompatibility rejection signaling. Notably, functional evidence supporting these modifiers varies substantially across plant families and genetic backgrounds. In this section, these regulatory factors are clearly classified into two categories: those with functional validation in Solanaceae (potato and related close relatives), and those inferred preliminarily from non-Solanaceae species. Each modifier is further contextualized according to the strength of available experimental evidence.

#### 2.3.1 HT-B proteins

HT-B proteins are stylar-specific cysteine-rich proteins with conserved, essential roles in modulating *S-RNase* stability and cytotoxicity within the GSI signaling pathway. The core regulatory functions of *HT-B* are well-characterized and experimentally validated in potato and other Solanaceae species. Functional studies confirm that *HT-B* directly interacts with *S-RNase*s in stylar transmitting tissues, regulating their accumulation, protein stability and intracellular localization in pollen tubes (Goldraij et al., 2006; Tovar‐Méndez et al., 2017). Silencing or mutation of *HT-B* weakens SI rejection, attenuates *S-RNase*-mediated pollen tube growth arrest, and promotes SC transition in Solanaceae crops. Owing to robust experimental support in potato and related Solanaceae materials, *HT-B* represents a high-confidence modifier for SC regulation and potato breeding.

#### 2.3.2 Thioredoxins

Thioredoxins are key redox regulators maintaining protein conformation and redox homeostasis during plant reproductive development. Functional evidence is partially characterized in Solanaceae model species, while most regulatory mechanisms are inferred from cross-species conserved pathways. Thioredoxins can modulate the redox state of *S-RNase*s and downstream signaling components, mitigating the cytotoxic effects of self *S-RNase*s on pollen tubes and alleviating SI responses (Cabrillac et al., 2001). Although overexpression and physiological assays support their regulatory roles in SC, targeted gene editing and transgenic validation in potato remain limited. Accordingly, thioredoxins are regarded as intermediate-confidence SC modifiers with partial Solanaceae experimental support.

#### 2.3.3 Calcium signaling pathway components

Calcium signaling components, including calcium channels and calcium-dependent protein kinases, act as core second messengers governing pollen tube growth, polarity establishment and stress responses. Regulatory roles in SI/SC are largely putative, with evidence mainly derived from non-Solanaceae species; direct functional validation in potato is still lacking. Calcium homeostasis alterations influence pollen tube cytoskeleton organization and growth behavior, thereby indirectly modulating the intensity of SI rejection signaling (Yang et al., 2021). To date, only correlative physiological evidence is available, without targeted gene knockout or transgenic verification in potato. Thus, calcium signaling components are defined as low-confidence candidate SC modifiers requiring further experimental validation.

In summary, these SC modifiers differ markedly in reliability and application potential according to taxonomic origin and experimental evidence strength. Well-characterized modifiers with solid Solanaceae support can serve as effective targets for potato SC molecular breeding, while intermediate and low-confidence factors require further functional validation in potato and Solanaceae germplasm.

## 3 Strategies to overcome self-incompatibility

### 3.1 Introgression of *Sli* via conventional and molecular breeding

Introgression of the functional *Sli* allele into elite diploid potato germplasm has emerged as one of the core prevailing strategies to break the inherent GSI barrier and promote the transformation of potato from heterozygous clonal breeding to diploid inbred hybrid breeding (Eggers et al., 2021; Ma et al., 2021). This strategy integrates conventional crossing procedures with modern molecular marker-assisted selection (MAS), which can efficiently introduce dominant SC traits into excellent SI backbone breeding materials while maintaining the excellent agronomic comprehensive traits of elite germplasm. In the actual breeding operation, the standard implementation process is to select stable SC donor materials carrying functional *Sli* alleles to hybridize and backcross with high-yield, high-quality and disease-resistant elite SI diploid parental lines, and rely on molecular marker tracking technology to stably screen and identify individual plants carrying the target *Sli* allele in segregating progeny populations, so as to quickly purify the genetic background and eliminate redundant unwanted exogenous genetic fragments (Hosaka and Hanneman, 1998b; Birhman and Hosaka, 2000; Ames et al., 2024). The *Sli* locus has been finely mapped to a 333 kb region on chromosome 12, and its widespread distribution in cultivated potato germplasm has been systematically validated (Clot et al., 2020).

Diagnostic genotyping markers represented by kompetitive allele-specific PCR (KASP) assays have further substantially improved the accuracy, high-throughput and efficiency of molecular marker-assisted breeding for *Sli* introgression, and become the preferred typing tool for potato SC directional breeding in recent years. Compared with traditional gel-based molecular markers with low efficiency and poor repeatability, KASP markers can realize rapid allelic discrimination of *Sli* locus in large-scale breeding populations, accurately distinguish homozygous dominant, heterozygous and homozygous recessive genotypes at the seedling stage, though homozygous SC genotypes show better phenotypic consistency than heterozygous ones (Kaiser et al., 2021), effectively avoid the waste of breeding resources caused by blind selfing of untyped materials, and accelerate the breeding cycle of SC improved lines. At present, this mature molecular breeding approach has been increasingly adopted for the rapid creation and stable cultivation of new SC diploid potato lines, and on this basis, continuous artificial selfing and successive generation purification can be carried out to gradually obtain genetically stable, uniformly agronomic and highly homozygous inbred lines suitable for subsequent hybrid combination preparation, though the process is often hampered by cumulative inbreeding effects.

However, in the actual process of inbred line continuous purification and breeding promotion, the wide application of Sli introgression breeding is still severely constrained by the ubiquitous adverse effects of inbreeding depression in diploid potato. Long-term continuous selfing will lead to the continuous homozygosis of a large number of recessive deleterious genetic variations inherent in potato germplasm, resulting in a series of adverse phenomena such as significant plant growth vigor decline, floral organ development abnormality, pollen fertility reduction, tuber yield and quality deterioration (Wu et al., 2023). In addition, segregation distortion occurs not only on chromosome 12 but also across the whole genome during inbreeding (Birhman and Hosaka, 2000; Kaiser et al., 2021), leading to severe individual selection attrition and elite line elimination, resulting in many excellent preliminary SC materials being eliminated in the middle breeding stage and failing to finally form stable available inbred lines. Moreover, although KASP marker-assisted selection can accurately screen individual plants carrying *Sli* allele at the genotypic level, it cannot fully capture the subtle phenotypic variation and comprehensive genetic background differences caused by polygenic regulation and epigenetic effects. Therefore, systematic phenotypic identification and field verification of SC characteristics and agronomic traits are still indispensable key links in breeding, which can effectively ensure the breeding quality and practical application value of newly developed diploid inbred lines.

### 3.2 Breaking self-incompatibility by genome editing

With the rapid development of precision genome editing technologies, CRISPR-Cas9-mediated targeted knockout has emerged as a promising and increasingly effective strategy to break SI in diploid potatoes, serving as a valuable complement to conventional breeding approaches. This method directly targets core genes in the GSI signaling pathway, mainly the *S-RNase* gene (the female specificity determinant of SI) and the *HT-B* gene (a critical modulator of *S-RNase* cytotoxicity). Genome editing enables direct modification of elite SI germplasm without repeated crossing and phenotypic selection, greatly shortening the breeding cycle and effectively avoiding linkage drag that commonly occurs in conventional introgression breeding.

Genome editing serves as a reliable alternative tool for engineering self-compatibility in diploid potato. Ye et al. (2018) firstly realized stable self-compatibility via targeted knockout of *S-RNase*, laying a foundational framework for subsequent editing-based SI modification. Functional studies further validated that CRISPR-Cas9-mediated disruption of either *S-RNase* or HT-B can effectively convert SI diploid lines to SC phenotypes (Goldraij et al., 2006; Enciso-Rodriguez et al., 2019; Lee et al., 2023). Current trials have generated SC phenotypes under tested controlled conditions, with no obvious adverse effects reported in the cited studies on major agronomic performance. Nevertheless, single-environment phenotypic evidence remains insufficient to confirm long-term field stability and wide adaptability of edited lines.

Several practical and technical constraints still limit potential future breeding applications of genome editing in potato. Potential off-target effects remain a major genetic and biosafety concern, which cannot be completely eliminated even with optimized gRNA design and editing systems, and may induce unintended genomic variations and unpredictable phenotypic alterations. Furthermore, potato genetic transformation and plant regeneration efficiency are strongly genotype-dependent, restricting universal application across diverse breeding germplasm. In addition, regulatory frameworks governing genome-edited crops differ substantially among countries and regions, resulting in inconsistent biosafety supervision and commercialization standards. Overall, although genome editing provides a promising complementary approach to conventional and marker-assisted breeding, its practical deployment in potato genetic improvement is still at an exploratory developmental stage, with large-scale commercial breeding application currently remaining limited.

## 4 Innovative paradigms of diploid hybrid potato breeding

The development of innovative diploid hybrid potato breeding systems relies fundamentally on the effective overcoming of self-incompatibility (SI) and the establishment of stable self-compatibility (SC). The breeding paradigms presented below are downstream or alternative strategies that rely on established SC but do not directly modify SI mechanisms.

### 4.1 Inbred line development and heterosis utilization

The establishment of SC represents an indispensable prerequisite for constructing homozygous inbred lines, which form the core parental foundation of diploid hybrid potato breeding (Jansky et al., 2016). Using SC germplasm derived from *Sli* introgression or CRISPR-mediated modification of SI components, breeders can progressively fix homozygosity and develop stable inbred lines with desirable agronomic traits. High-throughput KASP markers closely linked to the *Sli* locus further enable rapid and accurate selection of SC individuals during inbred line development (Sood et al., 2024).

In addition to traditional selfing-based purification, haploid induction and doubled haploid (DH) technology can be employed as a downstream auxiliary approach to accelerate inbred line development. Unlike strategies that directly modify SI genes to achieve SC, this technology does not alter the intrinsic SI mechanism but enables rapid generation of fully homozygous diploid lines (Yang and Zhou, 1982; Amundson, 2021; Patel and Jha, 2025). Anther culture represents a widely used *in vitro* method for haploid and DH production in tetraploid potato, enabling efficient regeneration of ploidy-variant plantlets (Zhang et al., 2023). DH technology can only be effectively utilized after the SI barrier has been overcome through Sli introgression or genome editing, and it replaces multiple generations of laborious continuous selfing to accelerate genetic background purification. DH lines are genetically stable and phenotypically uniform, providing ideal parental materials to ensure consistent agronomic performance of hybrid F1 progeny. Nevertheless, DH application still faces substantial constraints, including strong genotype-dependent induction efficiency, unstable chromosome doubling success rates, poor post-doubling fertility recovery, and inevitable exposure of deleterious recessive alleles after full homozygosity fixation.

Crosses between genetically divergent inbred lines can generate F_1_ hybrids with significant heterosis, often reflected in enhanced stress resistance, more uniform tuber traits, and broader environmental adaptability (Bachem et al., 2019; Zhang et al., 2019). Maximization of heterosis largely depends on genetic complementarity between parental lines, which requires the rational selection of SC lines with compatible genetic backgrounds and complementary phenotypic characteristics.

### 4.2 Self-incompatibility recycling for hybrid purity

SI recycling represents a promising strategy for improving hybrid TPS production efficiency by reducing reliance on manual emasculation. This system is built upon the restoration of SI in F_1_ hybrids through the segregation of S-haplotypes derived from two SC parental lines. The re-established SI in hybrids effectively prevents self-pollination and promotes outcrossing, thereby improving hybrid seed purity without intensive manual intervention. Although this strategy shows potential for simplifying hybrid propagation, its stability and efficiency remain influenced by S-locus genotypes, genetic background, and environmental conditions, requiring further validation before large-scale application in potato breeding.

### 4.3 Integrated speed breeding and genomic selection

The integration of speed breeding, genomic selection (GS), and precision genome editing provides a promising framework for accelerating diploid hybrid potato breeding, all of which depend on the prior establishment of self-compatibility. Speed breeding shortens generation cycles by optimizing growth conditions, enabling more rapid generation advancement of SC materials. Genomic selection supports early and high-throughput prediction of complex traits, allowing more efficient screening of SC lines and promising hybrid combinations. When combined with targeted editing of SI-related genes, these tools can help streamline breeding pipelines; however, efficiency varies across genetic backgrounds, and long-term field performance remains to be fully validated (Xu et al., 2022).

### 4.4 Alternative reproductive strategies: apomixis

Apomixis is an asexual reproductive system that produces clonal seeds without fertilization, representing an alternative reproductive strategy that reduces dependence on sexual reproduction and selfing rather than directly overcoming SI. Apomixis does not alter SI mechanisms but bypasses sexual reproduction entirely. In this context, apomixis serves not as a direct solution to overcome SI, but as a reproductive alternative that reduces dependence on selfing, *Sli* introgression, and annual hybrid seed production.

Conventional F_1_ potato hybrids exhibit strong heterosis, but sexual propagation leads to genetic segregation and progressive heterosis loss in subsequent generations. Apomixis can maintain hybrid genetic uniformity by producing clonal seeds identical to the maternal hybrid genotype, thereby stabilizing hybrid performance across generations. Functional apomixis requires the coordinated regulation of three key biological processes: apomeiosis, parthenogenesis, and autonomous endosperm development. The precise synchronization of these developmental events remains a major biological challenge for stable apomixis engineering in potato.

Recent studies have yielded preliminary exploratory progress in potato apomixis research, and the manipulation of core apomixis-related genes has successfully generated partial apomictic phenotypes in diploid potato germplasm (Mahlandt et al., 2023; Hojsgaard et al., 2024). However, fully stable, heritable, and agronomically functional apomictic potato lines have not yet been achieved, with critical issues regarding reproductive efficiency, genetic stability, and consistent heritability still unresolved. Compared with the well-characterized apomictic model *Hieracium*, potato apomixis research remains in the early exploratory stage, and further mechanistic studies are required to establish reliable apomictic systems for future diploid hybrid potato breeding.

## 5 Future perspectives

Future research directions toward diploid hybrid potato breeding should be rooted in a deeper understanding of SI mechanisms and the rational application of SC-related technologies. To achieve stable and efficient diploid hybrid breeding systems, research priorities should be balanced between practically achievable short-term goals and longer-term exploratory objectives.

In the short term, core efforts should focus on strengthening the efficiency and stability of SC induction systems mediated by *Sli* introgression and genome editing. It is critical to recognize that *Sli*-mediated SC is not universally effective across all genetic backgrounds, and its function may be modulated by additional genetic modifiers and environmental factors. This limitation is consistent with the variable performance of Sli-based SC observed in earlier germplasm evaluation and breeding application sections. Further characterization of these regulatory interactions will help improve the robustness and predictability of SC conversion in diverse elite germplasm. Additional short-term priorities include optimizing breeding pipelines for inbred line development, mitigating inbreeding depression and deleterious genetic load, and improving the reliability of phenotypic screening for SC traits. The integration of speed breeding and genomic selection can support more efficient generation advancement and early evaluation of SC materials, provided that these tools are calibrated to the genetic complexity of SI responses in potato. These breeding tools can only exert their full value on the basis of stable self-compatibility achieved by overcoming SI constraints.

In the longer term, more exploratory approaches such as apomixis engineering offer theoretical potential for clonal hybrid seed production, though stable and heritable systems remain unavailable. Research into apomixis should emphasize the coordinated regulation of apomeiosis, parthenogenesis, and endosperm development, while acknowledging substantial biological hurdles relative to well-characterized model systems. Apomixis acts as an alternative pathway to bypass SI barriers rather than directly manipulating SI genetic pathways. Expansion of diploid germplasm diversity will also facilitate the discovery of novel SI modifiers and compatibility factors beyond *Sli*, supporting more flexible and resilient hybrid breeding systems. Enriching germplasm resources further provides valuable genetic reservoirs for dissecting SI mechanisms and breeding new SC materials.

Overall, sustainable progress in diploid hybrid potato breeding requires mechanistic precision, balanced interpretation of emerging technologies, and clear prioritization between immediately actionable improvements and longer-term innovative goals.
